# Fault Diagnosis of Internal Combustion Engine Valve Clearance Using the Impact Commencement Detection Method

**DOI:** 10.3390/s17122916

**Published:** 2017-12-15

**Authors:** Zhinong Jiang, Zhiwei Mao, Zijia Wang, Jinjie Zhang

**Affiliations:** Diagnosis and Self-Recovering Engineering Research Center, Beijing University of Chemical Technology, Beijing 100029, China; jiangzn@mail.buct.edu.cn (Z.J.); 2015400122@mail.buct.edu.cn (Z.M); 2017200672@mail.buct.edu.cn (Z.W)

**Keywords:** valve clearance fault diagnosis, internal combustion engine, vibration signal processing, condition monitoring

## Abstract

Internal combustion engines (ICEs) are widely used in many important fields. The valve train clearance of an ICE usually exceeds the normal value due to wear or faulty adjustment. This work aims at diagnosing the valve clearance fault based on the vibration signals measured on the engine cylinder heads. The non-stationarity of the ICE operating condition makes it difficult to obtain the nominal baseline, which is always an awkward problem for fault diagnosis. This paper overcomes the problem by inspecting the timing of valve closing impacts, of which the referenced baseline can be obtained by referencing design parameters rather than extraction during healthy conditions. To accurately detect the timing of valve closing impact from vibration signals, we carry out a new method to detect and extract the commencement of the impacts. The results of experiments conducted on a twelve-cylinder ICE test rig show that the approach is capable of extracting the commencement of valve closing impact accurately and using only one feature can give a superior monitoring of valve clearance. With the help of this technique, the valve clearance fault becomes detectable even without the comparison to the baseline, and the changing trend of the clearance could be trackable.

## 1. Introduction

Internal combustion engines (ICEs), as a power source, are widely used in the automobile industry, ship and power equipment. However, the complex structure, and harsh and fickle working conditions usually result in unexpected faults. A serious failure of the ICE may cause loss of production or even human casualties. The techniques of condition monitoring and fault diagnosis have received increasing recognition and proven to be very valuable. Furthermore, the recent achievements of these techniques for industrial applications is well reviewed and summarized in [1,2,3]. The reported faults mainly include misfire, knock, piston slap, injection faults, valve fault, bearing cap with wear and connecting rod with un-correct screw preload, etc. [1]. Furthermore, the techniques for ICE fault detection mainly include vibration [4], acoustic emission [5], in-cylinder pressure [6] and the crank angular velocity [7] as well as oil [8], etc.

The abnormal clearance fault of an ICE may lead to a decline in the performance and reliability, as well as occurrences of malignant failure such as valve fracture and cylinder hit fault [9]. In fact, the valve train clearance, one of the critical motion mechanisms controlling the timing of gas intake and exhaust, is essential for thermal compensation. However, it frequently tends to increase due to wear of the components or a faulty adjustment during engine overhaul [10]. In order to identify the fault in time based on the signal analysis, and then to conduct maintenance at the early stages of the fault to avoid more severe deterioration and more economic losses, the research on detecting and even tracking the valve train clearance of an ICE is of great importance.

Several significant works on detecting the valve clearance fault have been reported. Vibration and acoustic emission analysis techniques are considered to be more suitable for implementation on on-board monitoring systems as their signal acquisitions are non-intrusive and convenient [11]. However, vibrations and acoustic emission signals generated by an ICE are highly transient and non-stationary, i.e., cyclostationary, and always inevitably contain the response of multiple exciting forces. These make it very difficult to extract fault-sensitive characteristics of significant importance for fault detection [12]. Therefore, many advanced signal processing techniques were proposed to extract fault features from ICE signals. In [13], an impact strength feature was generated for each valve closing impact by calculating the local root mean square and localizing the impact with adaptive thresholding. An EMD-based signal processing method was proposed to detect the occurrence of impacts generated by valve closing and piston slap in [12]. In [10], a set of features was extracted from vibration signals only in the time domain. With these features, four commonly used supervised classifiers were trained and tested. It was reported that the performance of classifiers would improve when more features were selected. In [14], a two-load acoustic method was developed to detect and diagnose the exhaust valve fault. The source strength characteristic extracted from the pressure waveforms was proved to be useful by conducting experiments on a four-cylinder diesel engine by enlarging the valve clearance from 0.35 mm (normal) to 0.7 mm and 1.7 mm. In [15], the exhaust valve clearance fault was introduced to one cylinder of a four-cylinder ICE, and the faulty condition was distinguished from the healthy condition in the time domain and frequency domain of acoustic emission signals. In [16], the problem of feature extraction was turned into an image classification problem. Firstly, vibration acceleration signals were transferred to time–frequency images by the Wigner–Ville distributions. Then, the probabilistic neural networks were directly used to perform a classification without extracting other fault features. With a similar idea to [16], cone-shaped kernel distributions and a neural network were used in [17] to diagnose the valve fault. Despite many recent studies on the feature extraction and fault diagnosis for the ICE valve clearance fault, practical applications in real-time monitoring are scarce, because most of these methods require a lot of data collected during the healthy condition to obtain the nominal baseline or data collected during the fault condition to test the fault classifier, all of which are very difficult to implement on an in-service engine.

In this work, we study the detection approach of the ICE valve clearance fault using vibration analysis. Vibration signals are measured on the engine cylinder heads of a 12-cylinder ICE. The measurement is conducted at three different fault levels, three different operating speeds and five different operating loads to provide investigative data. The main contribution of this work can be summarized as follows: (1) a fault feature which is sensitive to the valve clearance and insensitive to working conditions is selected through dynamic simulation; (2) a novel impact commencement detection method which is capable of obtaining a sufficiently accurate result is proposed; (3) based on experimental verification, using only one feature can provide superior monitoring and tracking of valve clearance without comparison to the baseline extracted during the healthy condition; and (4) there is potential for applying the fault diagnosis method in real-time mode since it has good adaptability and robustness.

The rest of this paper is organized as follows. The test rig and experiment procedure are described in Section 2. An elaboration based on dynamical simulation for the feature selection is presented in Section 3. Section 4 presents the proposed feature extraction approach. In this section, a signal pre-processing method based on filtering with adaptive cutoff frequency is presented, and a novel fault feature extraction approach based on an adaptive impact detection technique is also introduced. Section 5 reports the valve clearance fault detection results and verifies the validity of the diagnosis method. Finally, conclusions are presented in Section 6.

## 2. Diesel Engine Experimental Test System

### 2.1. Test Rig

All the experiments will be conducted on a TBD234 twelve-cylinder V-shaped direct injection diesel engine (Henan Diesel Engine Industry Co. Ltd., Luoyang, China). The major features of the ICE are summarized in Table 1. A hydraulic dynamometer is connected to the engine, so that different loads can be applied to it. The data used in this work are collected under variable working conditions.

**Figure 1:**
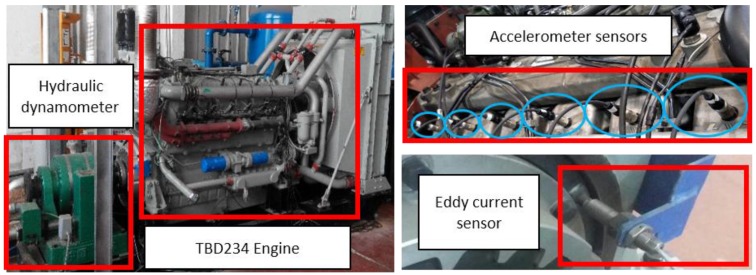
The Internal combustion engines (ICE) and sensors positioning.

**Figure 2:**
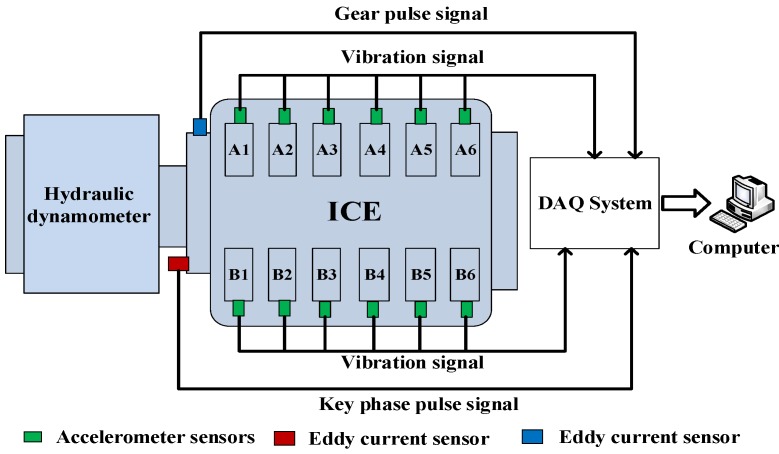
The interconnection of the main test rig components.

As shown in Figure 1, accelerometer sensors are mounted, using glue paste, on the upper surface of the cylinder heads to acquire vibration signals directly. In order to achieve the correlation between the vibration signals and the displacement of each cylinder piston, an eddy current transducer used for identifying the different phases of the working cycle is installed on the flywheel that is directly connected to the engine crankshaft. Only one marker was used per rotation for the eddy current sensor. Moreover, in order to calculate the crankshaft angular speed for translating signals from the time domain to the angle domain, another eddy current transducer is installed just beside the gear of the flywheel to capture the pulse signals generated by gears. The parameters of the accelerometer and the eddy current sensor are listed in Table 2 and Table 3, respectively. The accelerometer and pulse signals are sampled using a data acquisition (DAQ) system, in which the DAQ card has a 16-bit ADC resolution and a maximum sampling rate of 102.4 KS/s per channel, and up to 32 analog inputs. Signals are processed using a computer with 16 GB of RAM, and a 3.10 GHz Intel i7 processor. A photograph of the ICE and positioning of the sensors is shown in Figure 1 and the interconnection of the main test rig components is presented in Figure 2.

### 2.2. Introduction to the Valve Train Faults of ICE 

**Figure 3:**
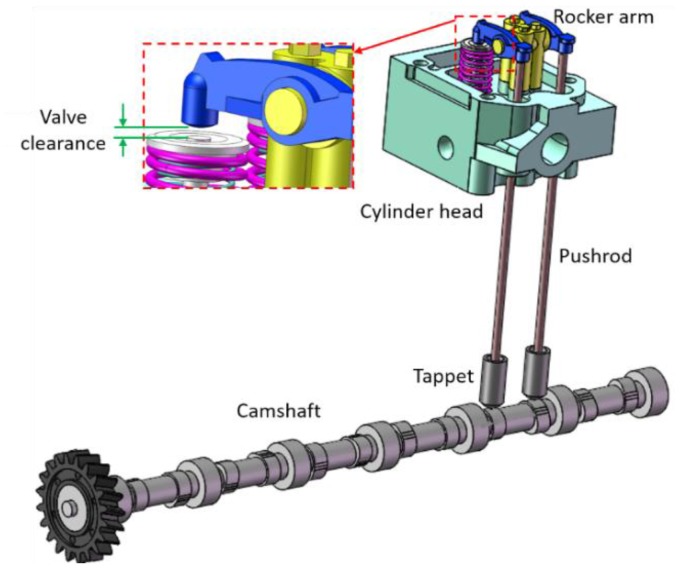
The solid model of the valve train.

In the ICE, the valve train controls the flow into and out of the combustion chambers. Figure 3 depicts the solid model of the valve train established based on the test rig, which typically consists of one camshaft, two tappets, two pushrods, two rocker arms, intake and exhaust valves, and valve springs. The valve clearance refers to the distance between the valve cap and rocker as illustrated in Figure 3. In the valve train system, the camshaft driven by the crankshaft controls the valve behaviors, and the lifting of the valve is determined by:

(1) $$l_{VAL}(\theta )=l_{CAM}(\theta )\cdot \lambda_{RA}-c_{VT}\;-\;\frac{F_{pre}}{K_{VT}}$$

where θ is the crank angle, $l_{CAM}(\theta )$ is the lifting of cam, $\lambda_{RA}$ is the arm length ratio of the rocker, $c_{VT}$ represents the valve clearance, $F_{pre}$ is the pretension force of valve springs, and $K_{VT}$ represents the stiffness of the valve train. For a given ICE, the structural parameters, including the cam profile, $\lambda_{RA}$, $F_{pre}$, and $K_{VT}$, have been determined. From Equation (1), we can find that an abnormal valve clearance may lead to improper valve timings and then result in the engine performance being degraded. 

The engine’s manufacturer provides the normal limits of valve clearances or the valve timings. In the normal state of the TBD234V12 diesel engine (Henan Diesel Engine Industry Co. Ltd., Luoyang, China), the clearance should be between 0.25 and 0.35 mm for the intake valve, and between 0.45 and 0.55 mm for the exhaust valve. The chosen clearance fault levels of the exhaust valve are 0.8 mm and 1.1 mm, i.e., the values of 0.5 mm, 0.8 mm and 1.1 mm will be used for the normal and fault cases studied in this paper.

### 2.3. Test Procedure

Three sets of measurement experiments were carried out. The first set involves testing at 1500 rpm engine speed at three different engine loads: 700, 1000 and 1300 N m, and testing at 1800 rpm at four loads: 700, 1000 1300 and 1600 N m, and testing at 2100 rpm at five loads: 700, 1000 1300 1600 and 2200 N m during the healthy condition. The second and third sets of measurements are for the fault conditions with the valve clearance in the exhaust valve of the B1-cylinder of 0.8 mm and 1.1 mm in the same operating conditions as the first set. The asynchronous sampling method is adopted, while all channels are kept in synchronization, and data was collected at the sampling frequency of 51.2 kHz per channel in all tests.

## 3. Feature Selection

Feature selection is a key step in the processes of fault diagnosis and prediction. It is generally related to two important aspects: firstly, the selected features should be sensitive to target faults and not sensitive or less sensitive to noises; then, the available signal processing techniques have the ability to extract the selected features with sufficient accuracy, satisfying the requirements of the engineering application. The objective of this work is to develop a method to detect and diagnose the abnormal ICE valve train clearance. The ideal selected features should be sensitive to abnormal valve train clearance and stable to engine operating conditions, because it is usually difficult to achieve the engine loads except for engines used for power generating sets. In order to select good features for diagnosing the valve clearance fault, studies on the influence of valve clearance on the kinetic and dynamic response of the valve train are conducted.

### 3.1. Valve Impacts Transmission Paths

**Figure 4:**
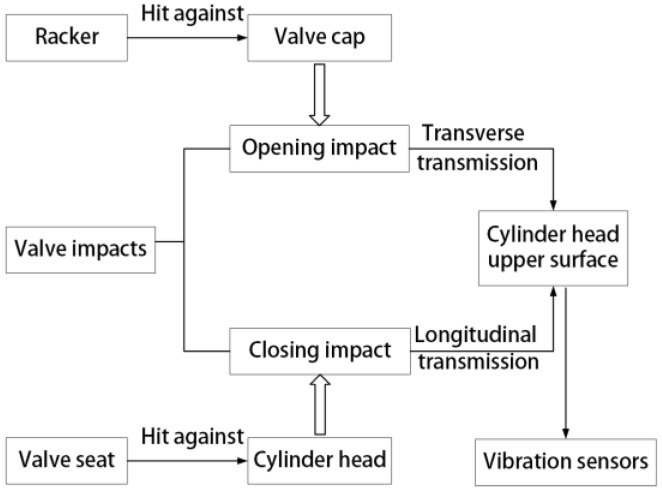
Valve impacts transmission paths.

Changing this clearance would have an influence on the kinematic and dynamic response of valve lifting. The valve impacts include an opening impact generated from the collision between the rocker and valve cap, and a closing impact generated from the collision between the valve seat and cylinder head. The vibration transmission paths are shown in Figure 4.

### 3.2. Simulation Study

To study the movement characteristics of the valve train mechanism, ADAMS, a multi-body dynamic simulation tool, is employed to simulate and to obtain the dynamic influence of the valve clearance and operating condition. The solid model used for simulation is shown in Figure 3, as previously mentioned. In order to keep the analysis simple and to illustrate the dynamic behavior, all the bodies are considered to be rigid. A contact pair model is introduced to simulate the clearance [18]. Two sets of simulations are performed: one is for a different valve clearance simulated by setting a different valve clearance, and the other one is for different operating conditions simulated by changing the engine speed.

**Figure 5:**
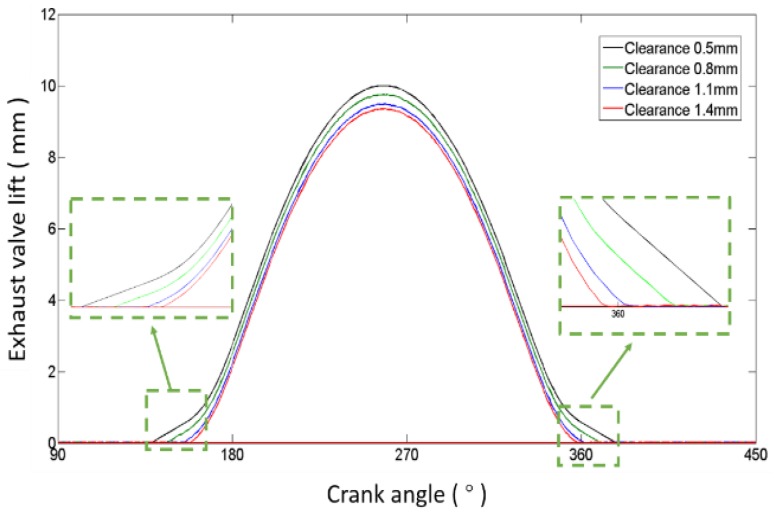
Valve lift of different clearances.

**Figure 6:**
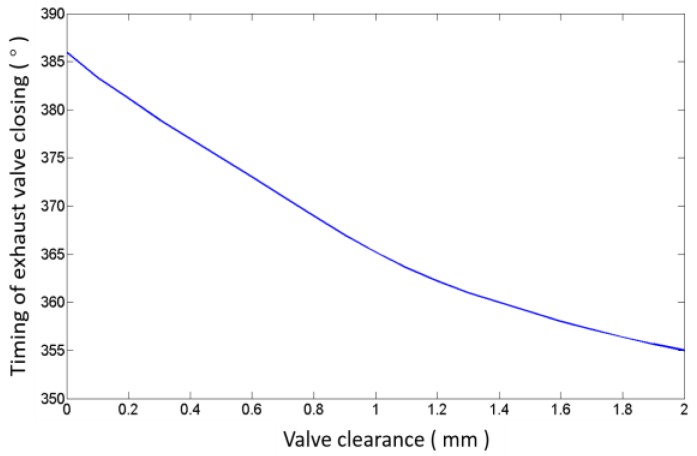
The relationship between valve clearance and the timing of valve closing.

**Figure 7:**
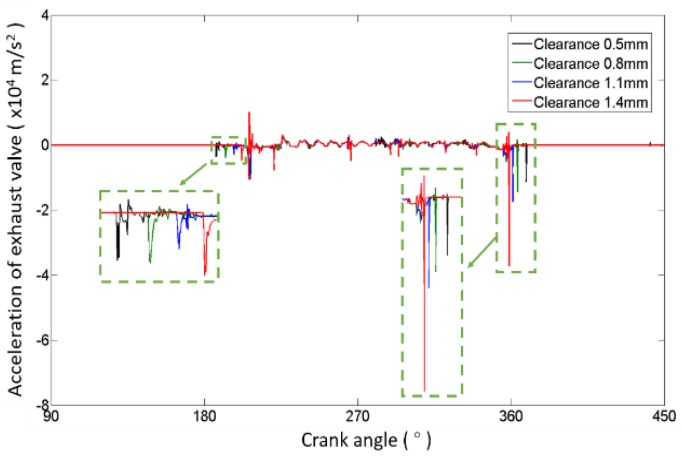
Valve acceleration of different clearances.

Four valve lift curves corresponding to clearances of 0.5 mm (normal), 0.8 mm, 1.1 mm and 1.4 mm are drawn in Figure 5, respectively. When the valve clearance increases, the time of valve opening is delayed and the time of valve closing is advanced. Because the buffer section of the cam is nonlinear, the changing degree of delaying or advancing becomes smaller when the clearance increases, as shown in Figure 6. The results can also be obtained from Figure 7, in which four valve acceleration curves, corresponding to four clearances, are shown. Meanwhile, Figure 7 shows that high impacts occur at the moments when the valve opens and closes; the closing impact is much stronger than the opening impact. Moreover, the impacts become stronger as the clearance increases. Combining Figure 5, Figure 6 and Figure 7 to analyze the characteristics of sensitivity to the valve clearance, we can ascertain that the timings of valve impacts have more of an effect than the strength of impacts when the clearance is small, but the result reverses when the clearance is large. This is due to the cam profile which contains the buffer section and rapid lifting section. Therefore, the timing of the impact is more capable of detecting the abnormal clearance in the early stage, and the valve closing impact is much easier to detect.

**Figure 8:**
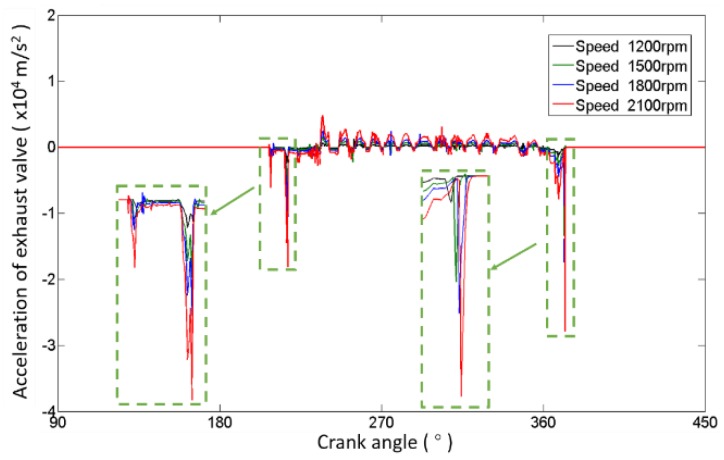
Valve acceleration of different speeds.

On the other hand, the simulation results corresponding to different operating conditions (1200 rpm, 1500 rpm, 1800 rpm, and 2100 rpm) with normal clearance are shown in Figure 8. It is obvious that the impacts become stronger with the engine speed rising, while the timing of impacts almost stays the same. The valve lift curves are not drawn because they are completely coincident.

### 3.3. Feature Selection

As shown in Figure 7 and Figure 8, the opening impact is usually very small and easily influenced by noise. So, the features extracted from valve closing impacts are much better to study for detecting the abnormal clearance in a valve train. Generally, the features related to valve closing impact can be divided into three categories: the impact strength features, the impact frequency features and the timing of impact appearance features. 

The first category includes features such as the peak and the peak-to-peak, the root mean square, etc. These features are extracted directly from the time domain and indicate the overall level, or the peakedness, or the flatness of the vibration signals. The simulation results show that the first category features are connected to the operating conditions. In particular, the valve closing peaks have unpredictable behaviors and even change considerably in a random manner. Therefore, the first category features may not be useful for the valve fault detection. 

The second category features are extracted from the vibration signal after transformation to the frequency domain usually by means of FFT. These features can often be effective for diagnosing the faults that may have detectable characteristic frequency components of the vibration signal. So, the application of the frequency domain is usually more successful for stationary vibration signals, such as the vibration signals of most rotating machines. However, the vibration signals measured on the upper surface of the cylinder heads are usually non-stationary signals resulting from the variation of the crankshaft angular speed of ICEs. According to previous studies [1,17] and the vibration tests on various ICEs, a typical frequency spectrum of cylinder head vibration, as shown in Figure 10 (the solid blue line), indicates that the frequency spectrum is chaotic and complex. Thus, it is difficult to use the frequency features for the valve fault diagnosis of ICEs.

The representative feature of the third category is mainly the timing of valve closing. The simulation results indicate that the valve would open late and close early when the valve clearance increases. In fact, the theoretical timing of valve closing is mainly determined by the cam profile, the arm length ratio of the rocker, and the valve train clearance. The lift of the valve train can be approximately derived based on these parameters, almost without consideration of the engine operating conditions. 

Therefore, we select the timing of valve closing as the target feature, i.e., the commencement phase of valve closing impact.

### 4. Feature Extraction Approach

#### 4.1. Signal Pre-Processing

##### 4.1.1. Angle Domain Translation Based on Soft Re-Sampling

To carry out the commencement phase of valve closing impact, the angle-invariant interval sampling mode, rather than the time-invariant interval sampling mode, is usually recommended to collect the vibration signals, in which the variations in ICE speed will have less impact on the measured signals. However, it is well known that the angle-invariant interval sampling approach requires a high-cost analog anti-aliasing filter, while the time-invariant interval sampling has relatively lower requirements and is simple to implement. Therefore, an indirect approach, combining time-invariant interval sampling with soft re-sampling, is employed in this study. 

The rationale and procedures of the introduced method can be found in [19,20]. Furthermore, the main procedures can be divided into three steps summarized as follows. Firstly, collect all the signals, including the vibration signal, the key phase pulse signal indicating the top dead center (TDC) of a specified cylinder, and the gear pulse signal used for calculating the crankshaft angular speed, at a fixed sampling rate simultaneously. Secondly, calculate the instantaneous rotation angle with the help of the gear pulse signal, and segment the signals into integral periods using the key phase pulse signal. Finally, re-sample the vibration signals digitally with a constant-angle interval using an interpolation method.

##### 4.1.2. Filtering Using Adaptive Cutoff Frequency

**Figure 9:**
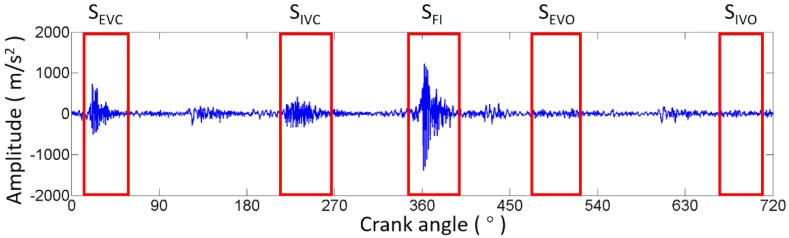
Raw vibration signal collected on the B1-cylinder head during one combustion cycle.

**Figure 10:**
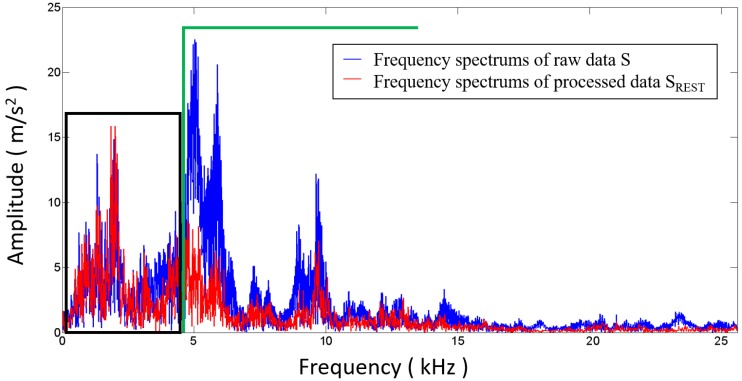
Comparison of the raw data *S* and processed data *S_REST_* in the frequency domain.

**Figure 11:**
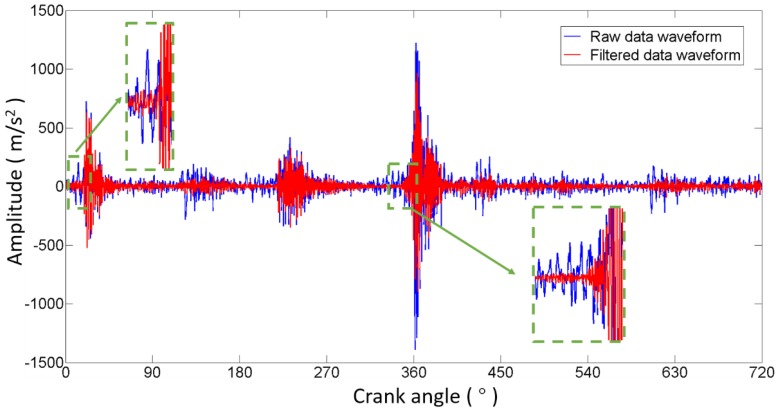
Comparison of the raw data and filtered data waveforms in the angle domain.

In order to improve the accuracy of fault detection and diagnosis methods, the vibration data should be filtered through a high-pass or band-pass filter with a certain cutoff frequency [13]. Considering that higher frequency may appear when the fault occurs and becomes worse, we employ a high-pass filter to ensure that failure information would not be lost. Generally, the cutoff frequencies are empirically selected or manually tried. However, for different ICEs, the target frequency ranges of the vibration response are different. Therefore, a method for choosing the cutoff frequency that is suitable for different ICEs is developed in this work. Firstly, the distinct impacts representing valve closing and opening, as well as combustion, are segmented. The ICE parameters, including firing sequence, injection timing, and valve timing, are used for identifying the range of specific impacts, i.e., the valve opening and valve closing impact regions are calculated according to the firing sequence and valve timing, and the fire combustion impact range is recognized based on the firing sequence and injection timing. The impact regions are extracted from the original angle waveform of the raw vibration signal cycle by cycle. We named the rest signals S_REST_. This step is described as follows:

(2) $$S_{REST}=S-S_{IVO}-S_{IVC}-S_{EVO}-S_{EVC}-S_{FI}$$

where S is the original angle signal, and $S_{IVO}$, $S_{IVC}$, $S_{EVO}$, $S_{EVC}$ and $S_{FI}$ represent the intake valve opening, intake valve closing, exhaust valve opening, exhaust valve closing, and fire combustion impact regions, respectively, as shown in Figure 9. Then, the frequency spectrums of S and $S_{REST}$ are calculated by means of FFT. As shown in Figure 10, the low frequencies of the spectrum of S are almost overlapped with the spectrum of $S_{REST}$, indicating that low frequencies are useless for studying the ICE abnormal clearance in the valve train. The cutoff frequency can be easily determined at the right edge of the overlap region. In Figure 11, the waveform represented by the solid red line is the data filtered through a high-pass filter with a cutoff frequency of 4.5 kHz, and the other waveform represented by the solid blue line is the raw data. The filtered waveform is similar to the raw one, but sharper in the transient response due to the removal of low frequencies in the high-pass process. The degree of sharpness is related to the components of low frequency noise around the transient impact. The advantages of the cutoff frequency determining method developed in this work are two-fold: firstly, it improves the filtering effect and lightens the workload of technicians, since it avoids manual selection by relying on experience or constant trials, and secondly, it is an adaptive method that is suitable for each ICE, theoretically. Furthermore, the cutoff frequency could be determined automatically by employing a recognition technique to identify the right edge of the overlap region.

#### 4.2. Impact Commencement Detection

It is well known that thresholding is a frequently used technique to detect the impact region in signal processing. In many cases, the threshold parameters are set manually, leading to risks of unreasonable setting. In this application, an adaptive method-based optimized maximum ascending gradient will be used to extract the start time of an impact. 

As an impact is the result of a sudden collision between components, the vibration energy will suddenly increase upon commencement of the impact. Then, it will decrease gradually due to the existence of structural impedance. Therefore, theoretically, the energy increase rate may be used to find the commencement of an impact, i.e., the maximum ascending gradient point of vibration energy can verify the impact location. However, the signal energy gradient is usually influenced by many factors. In this work, we further enhanced the impact detection method to overcome this problem by changing the policy of maximum point selection.

The main steps can be summarized as follows:

1. Calculate the local energy using a sliding window method. The local energy values were calculated by sliding a given window through the vibration signal and calculating the local energy for each data point of the signal. The process can be described mathematically as:
  (3) $$E(i)=\frac{1}{2N_{w}+1}(s{\left(i+N_{w}\right)}^{2}+s{\left(i+N_{w}-1\right)}^{2}+\ldots +s{\left(i-N_{w}\right)}^{2}),$$
  where s(i) is the given cycle vibration signal, and E(i) is the local energy, and $N_{ws}$ ($N_{ws}$ = 2 $N_{w}$ + 1) is the window width. $N_{ws}$ was related to the interval of the angle domain sampling or re-sampling. In this study, the constant-angle interval is a 0.2 crank angle, i.e., the re-sampling rate is 5 points per crank angle; the best value of $N_{ws}$ is empirically found to be 20.
2. Calculate the signal energy gradient. It is calculated with the following equation:
  (4) DE(i)= E(i+1)−E(i) ,
  where DE(i) is the local energy gradient.
3. Find the commencement of impact. We find the kth maximum value rather than the first maximum value in DE(i), and then look for the first point which is equal to or bigger than the kth maximum value, and consider that point as the commencement of impact. This step can be written mathematically as:
  (5) $$DE(u)\geq DE_{kth\max},$$
  (6) $$index_{SP}=u(1),$$
  (7) $$CI=DE(index_{SP}),$$
  where $DE_{kth\max}$ is the *k*th maximum value in DE(i), i.e., $k=num\{DE(i)\geq DE_{kth\max}\}$, and DE(u) is the sequence that is not less than $DE_{kth\max}$ in DE(i), $index_{SP}$ is the value of the first point in u(i), and CI is the commencement of the impact.

#### 4.3. Valve Closing Impact Commencement Extraction

The exhaust valve closing impact region was extracted from the filtered vibration signal in the angle domain. The segmental signal will be used in the subsequent analysis. 

**Figure 12:**
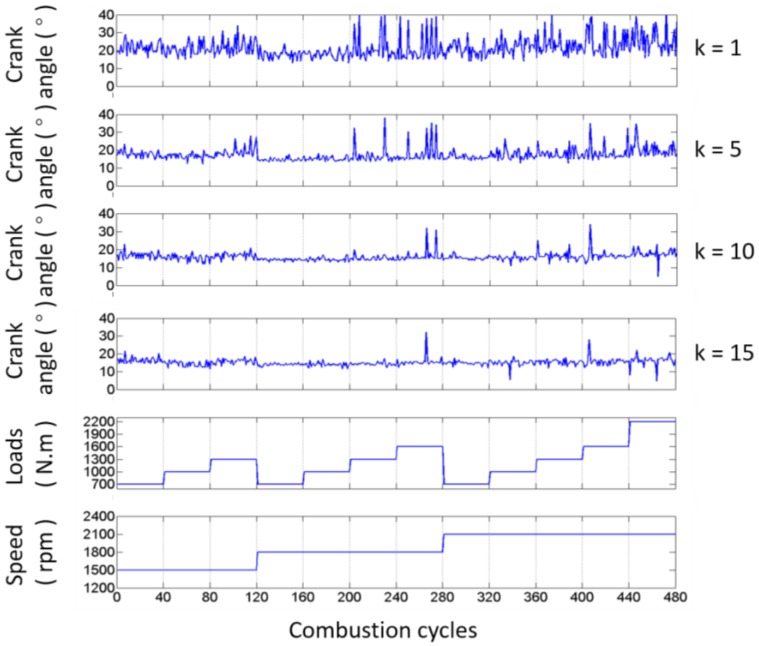
Angle and range fluctuations of the commencement of B1-cylinder exhaust valve closing impact in 480 combustion cycles with different values of k.

**Figure 13:**
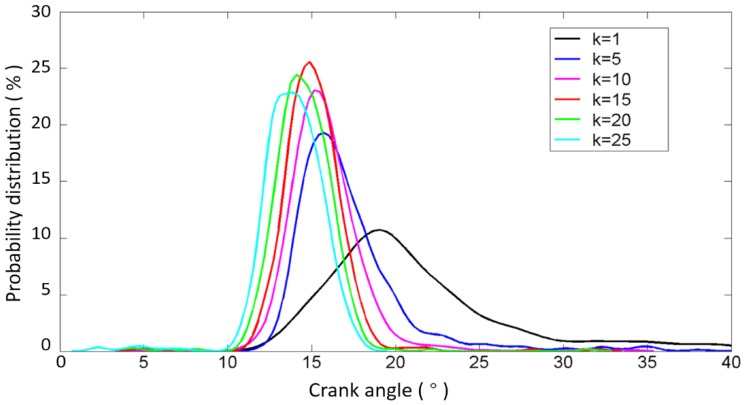
Probability distribution of the commencement of B1-cylinder exhaust valve closing impact in 480 combustion cycles with different values of k.

To confirm the ability of the impact detection method presented in this paper, the test results of the exhaust valve closing commencement phase with different values of k introduced in Equation (5) are shown in Figure 12, based on the data from the B1-cylinder head sensor shown in Figure 1 measured when the engine was in a healthy condition. Meanwhile, the fluctuations of the angle ranges are also displayed against 480 combustion cycles (40 combustion cycles per operating condition) in Figure 12. It is shown that the mean value and angle range fluctuation corresponding to each value of k are different. The mean values, the variance values, and the number of outliers corresponding to more values of k are tabulated in Table 4. Figure 13 shows the probability distribution of the commencement of valve closing impact in 480 combustion cycles with different values of k. The mean value decreases, the variance value decreases first and then increases, and the number of outliers that are more than three times the standard deviation of plus (or minus) decreases (or increases) as k increases. This is due to the points in front being more likely to be identified when the value of k increases. On the other hand, the mean value of the valve closing impact phase extracted from the real response signature cycle by cycle is basically consistent with the theoretical occurrence time of the valve that is listed in Table 1 (13° ± 2° after TDC). The error is in the allowable range, i.e., less than 3 crank degrees, when k is greater than 10. The error decreases when a bigger value of k is selected. The calculation error may be due to the measurement error of the TDC signature and errors between the designed and real parameters of the ICE.

In addition, it is also worth noting here that the basic trend of the valve closing phase does not change with the working condition of the engine, as alluded to in the aforementioned analysis.

From Figure 12 and Table 4, it can be seen that the outliers always exist, due to the fact that the dynamic status signals of ICE cylinder heads are non-stationary signals because the running process of an ICE is usually non-stationary, as well as the influence of noises. However, since the outliers will have a negative effect on the fault diagnosis, their number is expected to be minimized. Hence, further optimization is employed to enhance the method by improving its robustness and applicability.

Note that changing the valve clearance is usually a slow process rather than an instantaneous one, and the valve closing phase is stable when the valve clearance remains unchanged, as previously mentioned. According to the three-sigma rule, singular measurement data can be suggested as an outlier if its error is three times greater than the standard error; singular data can be reasonably deleted and substituted with an arithmetic average value. As the error processing criterion is very simple to implement and widely used in practical engineering applications, it was introduced in this work to enhance the robustness. We used a given detection window to slide through the time series of the valve closing phase extraction results. The value of each point is calculated using:

(8) $$y(i)=\{\begin{array}{ll} y(i)\;, & \;if\;y(i)\in [\mu (i)-3\sigma (i),\mu (i)+3\sigma (i)]\\ \mu (i)\;, & \;if\;others \end{array},$$

where y(i) is the calculation result of the valve closing phase for each combustion cycle, and μ(i) is the local mean value calculated by Equation (8), and σ(i) is the local standard deviation determined using Equation (9):

(9) $$\mu (i)=\frac{1}{2Lw+1}\sum_{j=i-Lw}^{j=i+Lw}y(j),$$

(10) $$\sigma (i)=\sqrt{\frac{1}{2Lw+1}\;\sum_{j=i-Lw}^{j=i+Lw}\;{\left[y(j)-\mu (i)\right]}^{2}},$$

where Lws(Lws=2Lw+1) is the detection window width, and represents the number of cycles taken into account. In fact, the signal processing process is just like a moving filter which will introduce a short time delay. The maximum delay time can be calculated by:

(11) $$T_{D}=\frac{120Lw}{n}\;(s),$$

where $T_{D}$ represents the maximum delay time, and n is the average speed of an ICE. In practice, a balance between the outlier detection effect and the delayed imposition should be taken into account, since a small Lw cannot achieve a good detection effect and a large Lw will add a significant time delay to the fault diagnosis. The delay time of one cycle is 0.08 s when the ICE is running at 1500 rpm. In this work, we empirically selected the value of Lw to be 20 after performing a comparison of various Lw values. 

**Figure 14:**
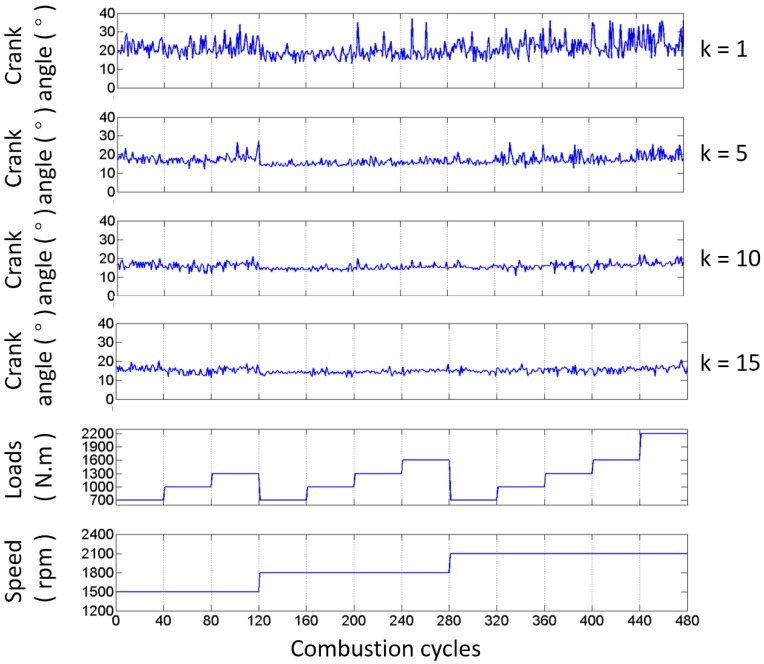
Angle and range fluctuations of the commencement of B1-cylinder exhaust valve closing impact in 480 combustion cycles calculated by the further enhanced method with different values of k.

**Figure 15:**
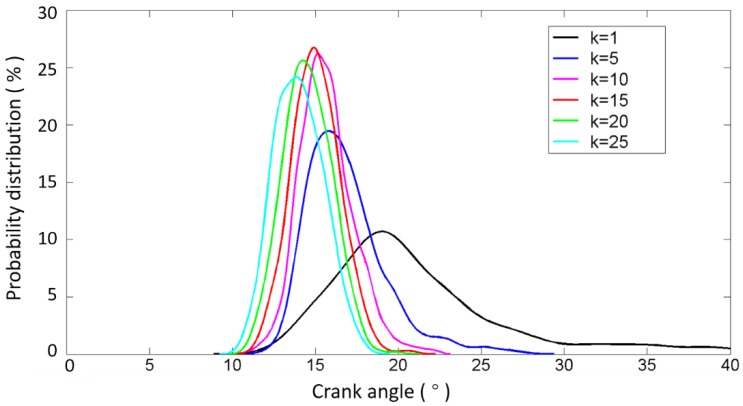
Probability distribution calculated by the further enhanced method to identify the commencement of B1-cylinder exhaust valve closing impact in 480 combustion cycles with different values of k.

The optimized results are shown in Figure 14 and Figure 15 as well as Table 5. It can be seen that such optimization steps would tend to make the fluctuation of the angle range more stable. The results show that the variation in the angle range decreases first and then increases as the value of k is increased. It is worth noting here that the probability distributions of the commencement of valve closing impact are similar and approximate when k is greater than 10. Thus, the selection of the value of k is not very strict, i.e., we may select k from a wide band without seriously affecting the accuracy of the results, which is of great significance for the applications. In the follow-up work of this paper, the value of k is selected as 15, since the variance becomes minimal when k is equal to 15, as shown in Table 5 and Figure 15.

## 5. Valve Clearance Fault Detection

In order to evaluate the performance of the feature extraction method for valve clearance condition monitoring, the abnormal valve clearance fault is introduced into the B1-cylinder, while the other cylinders are kept in normal condition. Three different valve clearances—0.5 mm (health condition), 0.8 mm and 1.1 mm (fault conditions)—are used to examine the fault diagnosis and prediction capabilities. To investigate the influence of the operating condition on the detection results, data from all sensors mounted on the engine was collected with three different operating speeds and five different operating loads for each valve clearance condition, as mentioned in Section 2.2.

**Figure 16:**
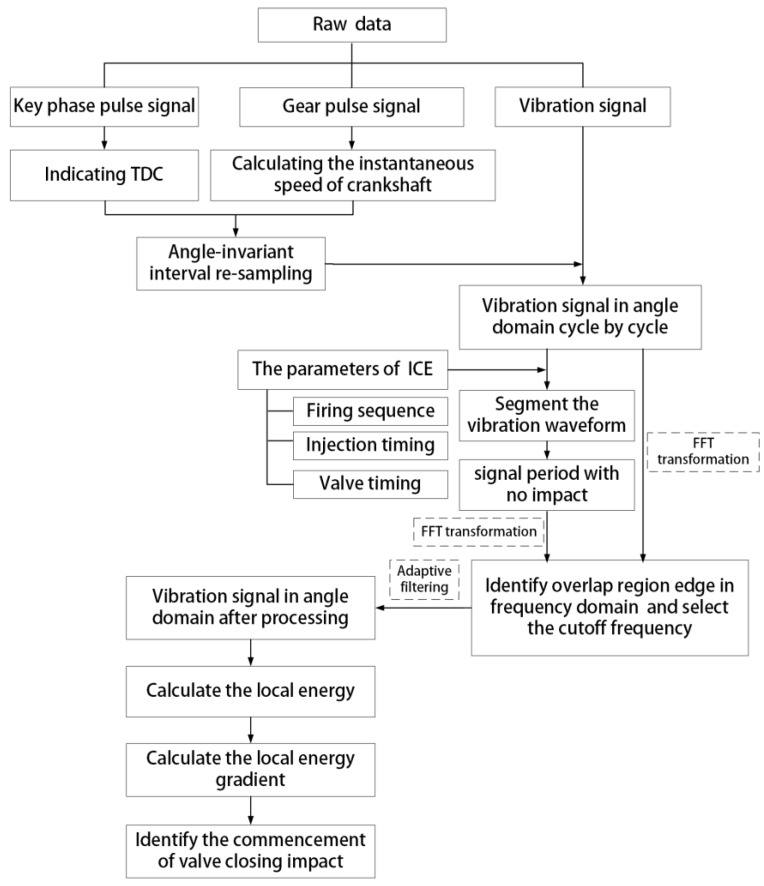
The procedures of the proposed method.

**Figure 17:**
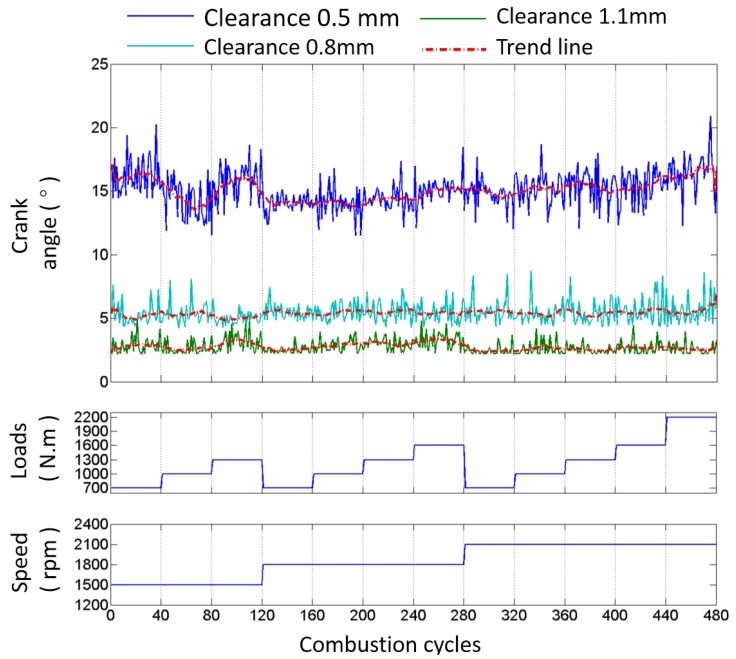
Angle and range fluctuations of the commencement of B1-cylinder exhaust valve closing impact in 480 combustion cycles calculated by the presented method with different valve clearances.

**Figure 18:**
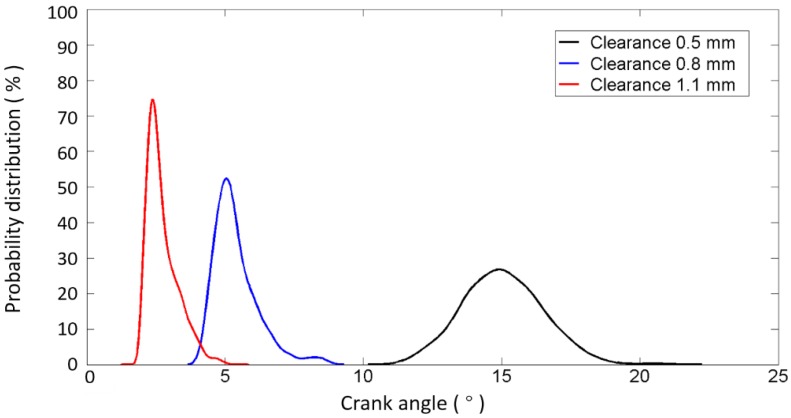
Probability distribution calculated by the presented method to identify the commencement of B1-cylinder exhaust valve closing impact in 480 combustion cycles with different clearances.

The procedures are briefly summarized in the flow chart presented in Figure 16, as mentioned above. The extraction results of the exhaust valve closing phase of the B1-cylinder at different valve clearances and different operating conditions are shown in Figure 17. The results show that, for each valve clearance, the valve closing phase is basically located in a small limited angle range even when the operating speed or load is constantly changing. This proves that the valve closing phase is not affected by variation in the operating condition. From Figure 17, it can also be seen that the commencement of valve closing impact obviously advances when the valve clearance increases. The difference in the advance is about 8° when the clearance increases 0.3 mm, and it becomes 10° when the clearance increases 0.6 mm. This not only proves the effectiveness of the presented method in detecting the valve clearance condition, but also verifies the importance of the phase extraction accuracy because the advance is not large enough. Meanwhile, it is worth noting that with the same 0.3 mm increase of valve clearance, the phase advance in the small clearance state (0.5 mm) is greater than in the large clearance state (0.8 mm), i.e., the phase advance has a nonlinear relationship with the increase of the valve clearance and is more sensitive when the clearance is small. This has a strong relationship with the cam profile and agrees with the simulation results. Moreover, Figure 18 shows the probability distribution of the commencement of valve closing impact in 480 combustion cycles with different clearances. It is easy to see that the data concentration becomes higher with the increase of valve clearance.

Hence, our accurate extraction method for the valve closing phase has been proven to be effective, and the fault detection performance based on it has been verified as good enough, as the valve closing phase is sensitive to changes in valve clearance and insensitive to the working condition of the engine.

## 6. Conclusions

In this study, an effective approach has been developed to detect and diagnose the ICE valve train clearance based on the vibration signal measured on the engine cylinder heads. This presented method is capable of monitoring the clearance accurately with only one feature—the commencement of valve closing impact—since the feature is sensitive to the valve clearance and insensitive to working conditions. Moreover, because this feature has clear physical significance, the baseline acquired during the healthy condition is no longer needed. Subsequently, a novel feature extraction technique was carried out based on a newly proposed impact detection method to meet the feature calculation accuracy requirement. The experimental results proved that the selected feature is indeed ideal for this fault detection, and the feature extraction results are accurate enough. In addition, this method has potential for application in real-time and the trend of the valve clearance may become trackable, as the calculation processes are adaptable and automatic with good robustness, and the implementation is simple. In the future, the new impact detection method will be extended to diagnose other impulse faults, and the valve clearance detection approach will be further studied and finally built into a real-time monitoring system for an engine.

## Figures and Tables

**Table 1:** Features of the TBD234 diesel engine.

Item	Value
Number of cylinders	12
Shape	V-shaped 60°
Firing sequence	B1-A1-B5-A5-B3-A3-B6-A6-B1-A2-B4-A4
Rating speed	2100 rev/min
Rating power	485 kW
Intake valve train normal clearance	0.3 mm
Exhaust valve train normal clearance	0.5 mm
Intake valve opening timing	26.5° ± 2° before top dead center (TDC)
Intake valve closing timing	46.5° ± 2° after bottom dead center (BDC)
Exhaust valve opening timing	47° ± 2° before BDC
Exhaust valve closing timing	13° ± 2° after TDC

**Table 2:** Parameters of the accelerometer.

Performance Specification	Unit	Measurement Model 8711-01 Accelerometer
Voltage sensitivity	mV/g	10
Frequency range (±5%)	Hz	1–10,000
Frequency range (±10%)	Hz	0.4–16,000
Natural frequency	Hz	42,000
Amplitude range	±g pk	500
Residual noise	g rms	0.0003
Mechanical shock limit	±g pk	5000
Temperature range	°C	−55 to 125
Amplitude linearity	%	±1

**Table 3:** Parameters of the eddy current sensor.

Performance Specification	Unit	Bently 3300 XL 11 mm Proximity Transducer System
Incremental scale factor	V/mm	3.94
Frequency Response	Hz	0–8000
Temperature range	°C	0 to 45
Linear Range	mm	4

**Table 4:** Statistics of the commencement of B1-cylinder exhaust valve closing impact in 480 combustion cycles calculated with different values of k; N1 and N2 represent the number of outliers that are more than three times the standard deviation of plus and minus, respectively.

k	Mean	Variance	N1	N2
0	21.1938	29.9478	12	0
5	17.2107	11.8933	12	0
10	15.8125	5.1422	7	1
15	15.0819	3.7590	4	3
20	14.4608	4.0561	2	6
25	13.8538	4.1,000	0	8

**Table 5:** Statistics of the commencement of B1-cylinder exhaust valve closing impact in 480 combustion cycles calculated by the further enhanced method with different values of k.

k	Mean	Variance
0	20.7403	21.4636
5	16.8284	5.6607
10	15.6677	2.6582
15	15.0469	2.0952
20	15.1344	3.2264
25	14.5093	3.4426

## Abbreviations

**List of Symbols**			
θ	crank angle	μ(i)	local mean value
$l_{CAM}(\theta )$	lifting of cam	σ(i)	local standard deviation
$\lambda_{RA}$	arm length ratio of rocker	Lws	detecting window width
$c_{VT}$	valve clearance	$T_{D}$	maximum delay time
$F_{pre}$	pretension force of valve springs	n	speed of the ICE
$K_{VT}$	stiffness of valve train	S	original angle signal
$S_{REST}$	rest signals	$S_{IVO}$	intake valve opening impact regions
$S_{IVC}$	intake valve closing impact regions	$S_{EVO}$	exhaust valve opening impact regions
$S_{EVC}$	exhaust valve closing impact regions	$S_{FI}$	fire combustion impact regions
E(i)	local energy	Nws	window width
DE(i)	gradient of local energy	$DE_{kth\max}$	kth maximum value in DE(i)
DE(u)	sequence that not less than $DE_{kth\max}$ in DE(i)	$index_{SP}$	value of the first point in μ(i)
CI	commencement of the impact	y(i)	calculation result of valve closing phase for each combustion cycle
